# Seasonal migrations, body temperature fluctuations, and infection dynamics in adult amphibians

**DOI:** 10.7717/peerj.4698

**Published:** 2018-05-08

**Authors:** David R. Daversa, Camino Monsalve-Carcaño, Luis M. Carrascal, Jaime Bosch

**Affiliations:** 1 Institute for Integrative Biology, University of Liverpool, Liverpool, United Kingdom; 2 Department of Biogeography and Global Change, Museo Nacional de Ciencias Naturales, CSIC, Madrid, Spain; 3 Centro de Investigación, Seguimiento y Evaluación, Parque Nacional de la Sierra de Guadarrama, Rascafría, Madrid, Spain

**Keywords:** *Batrachochytrium dendrobatidis*, *Bufo spinosus*, Migration, Hibernation

## Abstract

Risks of parasitism vary over time, with infection prevalence often fluctuating with seasonal changes in the annual cycle. Identifying the biological mechanisms underlying seasonality in infection can enable better prediction and prevention of future infection peaks. Obtaining longitudinal data on individual infections and traits across seasons throughout the annual cycle is perhaps the most effective means of achieving this aim, yet few studies have obtained such information for wildlife. Here, we tracked spiny common toads (*Bufo spinosus*) within and across annual cycles to assess seasonal variation in movement, body temperatures and infection from the fungal parasite, *Batrachochytrium dendrobatidis (Bd)*. Across annual cycles, toads did not consistently sustain infections but instead gained and lost infections from year to year. Radio-tracking showed that infected toads lose infections during post-breeding migrations, and no toads contracted infection following migration, which may be one explanation for the inter-annual variability in *Bd* infections. We also found pronounced seasonal variation in toad body temperatures. Body temperatures approached 0 °C during winter hibernation but remained largely within the thermal tolerance range of *Bd*. These findings provide direct documentation of migratory recovery (i.e., loss of infection during migration) and escape in a wild population. The body temperature reductions that we observed during hibernation warrant further consideration into the role that this period plays in seasonal *Bd* dynamics.

## Introduction

Parasites pose major risks to humans and wildlife, but widespread seasonality in prevalence of parasitic infections indicate that those risks vary over the annual cycle. While seasonal infection dynamics may at times be associated with host demographic factors (e.g., seasonal fluctuations in birth rates and mortality), such patterns may also be a function of seasonal changes in within-individual infections resulting from seasonality in host life history characteristics. Migratory wildlife undergo seasonal changes in behaviour and physiology, traits which affect basic drivers of within-individual infection dynamics such as rates of exposure to infective stages, rates at which exposure leads to infection (i.e., susceptibility), rates of parasite growth following infection (Altizer et al., 2006; Daversa et al., 2017). For example, migratory movements of juvenile salmon reduce exposure to parasitic lice (Krkosek et al., 2007), and immunosuppression in birds during migration may cause seasonal fluctuations in their susceptibility to infection (Owen & Moore, 2008). Direct documentation of seasonal fluctuations in within-individual infections throughout the annual cycle is limited, however (but see Knowles et al., 2011; Van Dijk et al., 2014; Spitzen-van der Sluijs et al., 2017), likely because of the difficulty in tracking individual hosts and their infections across multiple seasons. Such information could identify hosts that contribute disproportionately to parasite maintenance, clarify host traits that are linked to long-term parasite persistence and aid disease mitigation efforts.

Host movements comprising seasonal migrations could bring changes in within-individual infection through, for example, changes in host social behaviours that influence rates of exposure or changes in habitat use that affect environmental conditions conducive to within-host parasite growth (Daversa et al., in press). Both increases and decreases in infection prevalence following migration are well-documented (Altizer, Bartel & Han, 2011; Bauer & Hoye, 2014). Those broad patterns may be indicative of hosts contracting infections or losing infections (migratory recovery) (Shaw & Binning, 2016) while migrating, but could also arise from processes that do not entail changes in within-individual infections. For example, reductions in infection prevalence following migration could be driven by infection-induced mortality during migration (migratory culling) or migratory individuals leaving high-risk sites before contracting infections (migratory escape) (Bartel et al., 2011; Altizer, Bartel & Han, 2011), while increases in infection prevalence following migration could arise from influxes of susceptible hosts into parasite rich habitats (Van Dijk et al., 2014). To date, direct evidence for migratory-induced decreases or increases in infection at the individual level is largely lacking (Shaw & Binning, 2016).

Physiological changes experienced by migratory hosts during the annual cycle may also contribute to seasonal infection dynamics because such changes may affect susceptibility and resistance to infection. In ectotherms for example, seasonal changes in ambient temperature elicit seasonal changes in body temperature. Studies of amphibians have shown that seasonal decreases in body temperatures compromise immune function (Raffel et al., 2015), and when these compromises coincide with periods of high exposure, increases in infection prevalence may be particularly likely. Reductions in body temperature may also activate dormant parasite stages acquired during previous seasons (Glyfe et al., 2000), which could trigger spikes in prevalence even when exposure is limited (Langwig et al., 2014). Alternatively, ectotherms also thermoregulate by adjusting behaviours (Richards-Zawacki, 2009) and may undergo fever in response to infection (Sauer, Sperry & Rohr, 2016), which could drive losses of infection.

We carried out a longitudinal study of migratory amphibians to assess individual variation in seasonal movement patterns, body temperature and infection from the pathogenic parasite, *Batrachochytrium dendrobatidis (Bd)*. *Bd* is a microscopic fungus that infects the keratinized skin cells of many amphibian species via free-living aquatic zoospores that encyst into reproductive sporangia on infected hosts (Piotrowski, Annis & Longcore, 2004). Surveys of non-migratory hosts have found that *Bd* infections vary seasonally (Retallick, McCallum & Speare, 2004; Kriger & Hero, 2007; Murray et al., 2009; Sapsford et al., 2015), which may reflect seasonal changes in environmental factors like air and water temperature (Retallick, McCallum & Speare, 2004; Kriger & Hero, 2007; Murray et al., 2009). Surveys of migratory host species, while extensive, have predominantly focused on breeding seasons in aquatic habitats (Pilliod et al., 2010; Muths, Scherer & Pilliod, 2011; Bosch et al., in press), providing insight into inter-annual patterns of *Bd* infection but limited information on seasonal infection dynamics.

We focus on adult spiny common toads (*Bufo spinosus*), a competent *Bd* host that exhibits a highly seasonal life history. Adult spiny common toads annually convene in ponds and lakes for one to three months during the summer to breed. *Bd* zoospores rely on moist environments and therefore infect pond-breeding amphibians like spiny toads during occupation of aquatic breeding habitats. Toads then migrate from breeding sites to terrestrial habitats used for foraging and winter hibernations (i.e., “post-breeding migrations”) until migrating back to breeding sites the following year (i.e., “pre-breeding migrations”) (Sinsch, 1988; Daversa, Muths & Bosch, 2012). Studies report conflicting evidence for the growth and persistence of *Bd* infections in amphibians occupying terrestrial habitats (Stockwell, Clulow & Mahony, 2010; Raffel et al., 2015; Daversa et al., in press), differences which may depend on the type of substrate (e.g., soil versus sand) occupied by hosts. Previous work has shown that *Bd* can survive temperatures up to 30 °C (Piotrowski, Annis & Longcore, 2004) and that *Bd* sustains growth between 2 and 26/27 °C, depending on the strain (Voyles et al., 2017). *Bd* infections should therefore be sustained over host body temperatures across that range. We first carried out mark-recapture surveys over eight breeding seasons to assess inter-annual variation in prevalence and intensity of *Bd* infections in adult spiny common toads during aquatic breeding seasons. During that time, we radio-tracked a subset of toads to assess how infection burdens change in toads during post-breeding migration and subsequent terrestrial phases. We also recorded body temperatures of a subset of toads for an entire annual cycle to characterize how temperatures fluctuate throughout the year.

## Materials & Methods

### Study site and species

Adult spiny common toads were studied at permanent ponds in Guadarrama National Park, Spain (41°N, 4°W, elevation: 1,800–2,430 m). We focused on five of the ponds (elevational range: 1,956–2,175 m a.s.l.) where common toads breed annually: Laguna Grande (LG; elevation: 2,018 a.s.l), Laguna Chica (LCH; elevation: 1,956 m a.s.l), Laguna de Pájaros (LP; elevation: 2,175 m a.s.l.), Charca de la Mariposa (CHM; elevation: 2,136 m a.s.l.) and the Charca Larga y de las Piedras (CHLP; elevation: 2,110 m a.s.l.). The surrounding terrain consists of granite outcrops and alpine grasslands at higher elevations and heathland and pine forest at lower elevations. The park is the type locality of *Bd* in Europe, with first reports of chytridiomycosis in common midwife toads (*Alytes obstetricans*) in 2001 (Bosch, Martínez-Solano & García-París, 2001). *Bd* has also been detected in our focal host, but spiny toad populations have not suffered mass mortalities as midwife toad populations have (Bosch et al., in press). The costs of *Bd* to adult spiny toads are not well known, but experimental and field work have demonstrated that *Bd* infections can cause mortality in metamorphs (Bosch & Martínez-Solano, 2006; Garner et al., 2009; Bielby et al., 2015). The Consejería de Medio Ambiente of Madrid provided full approval for this research including fieldwork (10/025449.9/8, 10/168152.9/09,10/012157.9/10, 10/121009.9/11, 10/032921.9/12, 10/071126.9/13, 10/130923.9/14, 10/064263.9/15).

### Infection during aquatic breeding

We conducted mark-recapture capture surveys during toad breeding seasons (May–June) in 2008–2015 to determine infection status and intensity during aquatic breeding. We walked the perimeter of each pond at night and captured toads using dipnets. We recorded passive integrated transponder (PIT) tag numbers, and if animals did not already have a PIT tag, a new tag was inserted with a sterile syringe (Microplus, Insvet Inc., Esplus, Huesca, Spain) underneath the skin of the dorsal side. We then collected a sample of skin tissue by rubbing a sterile cotton swab (ref. 300261, Deltalab Inc., Barcelona, Spain), over the ventral side of the body and thighs (20 strokes) and the webbing of the hind feet (10 strokes), consistent with standard swabbing protocols (Briggs, Knapp & Vredenburg, 2010). Toads recaptured within the same breeding season were only swabbed once. Swabs were sprayed with 95% ethanol and stored refrigerated for a few weeks until processed.

### Infection during migration and terrestrial non-breeding seasons

In 2009 we installed transmitters in a subset of 20 toads at the end of the breeding season while toads were still occupying ponds. We attached transmitters (Bd-2 model, Holohil Systems Ltd., Canada) externally (*N* = 9 males and 2 females) or subcutaneously (*N* = 7 males and 5 females). Detailed information on our attachment procedures and transmitter specifications is reported in Daversa, Muths & Bosch (2012). The size of the transmitter was matched to the mass of the toad such that all transmitters weighed less than five percent of the body mass of the individual. Toads with both subcutaneous and external transmitters showed signs of normal behavior (e.g., burrowing in small rock crevices, undergoing amplexus).

We tracked toads throughout post-breeding migrations and subsequent terrestrial phases. We located toads once to twice per week between 0800 and 2,000 h using a TR-4 receiver (150/154 mH, Telonics, Inc., United States). If no signal was detected within the respective basin we attempted to obtain a signal in all adjacent basins. Once toads were located, individuals were hand-captured when possible (at times toads were burrowed under rock piles preventing capture). We identified recorded capture location using an eTrex HCx Global Positioning System (Garmin Ltd., Olathe, Kansas, USA). We then collected a tissue sample by rubbing a cotton swab as described before. We poured sterile water over the ventral side of terrestrial toads before swabbing because swabs must be moist to work effectively.

### Body temperature

In 2014 we subcutaneously implanted iButton Data Loggers (DS1922L; Maxim/Dallas Semiconductor Inc., United States) in a subset of 10 toads during the breeding season. All iButtons weighed less than five percent of the body mass of the individuals (>70 g) and toads showed signs of normal behavior after implantation. Toads were anaesthetized by immersion in a 0.4% aqueous solution of Tricaine methanesulfonate (MS-222, Sigma-Aldrich, Inc.) for implantation and removal and the skin was sutured with three surgeon’s knots using absorbable material. iButtons recorded body temperature every 2 h (i.e., 12 measurements per day) and their data were downloaded using Eclo ExpressThermo software (http://www.eclo.solutions/en/product_page/expressthermo).

### *Bd* detection

*Bd* DNA from swabs was quantified using standard realtime Polymerase Chain Reaction (qPCR) procedures (Boyle et al., 2004). We included amplification standards of 0.1, 1, 10 and 100 zoospore equivalents prepared from an isolate of known cell density (IA042, Spain) and a negative control in each plate. We used an internal positive control (IPC) to measure PCR inhibition in randomly selected samples that tested negative for *Bd* infection. Following the methodology of Hyatt et al. (2007), a VICTM-labelled synthetic amplicon was used as the IPC (VICTM dye, Applied Biosystems). The IPC was included in one of each duplicate well as 1 µl 10 × Exo IPCmix and 0.5 µl 50 × Exo IPC DNA. Infection loads are reported in zoospore equivalents (ZE), where one ZE is equivalent to a single zoospore. We considered ZE values of 0.1 or higher as positive for infection. All samples were analyzed in duplicate. Loads are reported as the mean and standard error, unless otherwise noted.

### Data analysis

We used mark-recapture data on toads captured at least twice to assess variability in *Bd* infections across survey years. We ran two generalized linear mixed effects models (GLMM), the first with infection status (0, 1) as a response and a binomial error structure, and the second with load (log-normalized ZE) as the response and a Gaussian error structure, as log-transformation of ZE values achieved normalization. In both models we included survey year as a fixed effect and PIT tag ID of toads as a random effect to account for repeated samples of individuals. For the models of infection intensity we only considered infected toads because we were interested in interannual variability in *Bd* loads among individuals testing positive for infection. We used likelihood ratio tests to determine the significance of inter-annual differences in infection prevalence and intensity by comparing models including year as a fixed effect with models omitting the effect.

A general linear mixed model was used to partition the variance in body temperature of toads according to sums of squares (SS) considering three independent components of variation: inter-individual differences (with individual toads as the levels of a random factor), within day changes (circadian; using time of day as a covariate) and year-round variation (employing the julian date as a covariate). Sample units were temperature measurements every two hours. To account for non-linear effects of time of day and julian date, we defined linear, quadratic and cubic polynomial terms of standardized predictors (i.e., at mean zero and sd = 1). Data were analyzed using StatSoft’s Statistica 10.0 (StatSoft Inc, Tulsa, Oklahoma).

## Results

### Infection during aquatic breeding seasons

**Figure 1:**
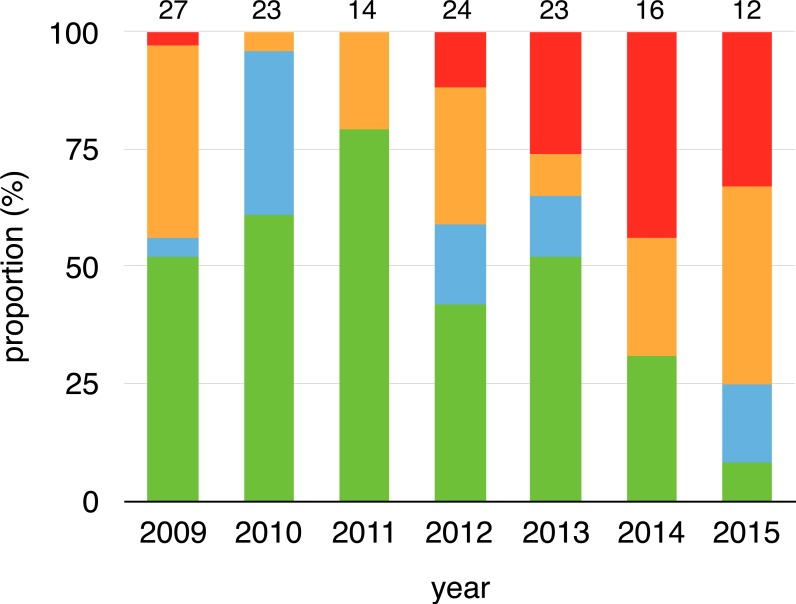
Changes on infection status of toads during aquatic breeding seasons from 2008 to 2015. Proportion of toads that remained positive (red), remained negative (green), changed from negative to positive (orange) or changed from positive to negative (cyan) for every year related to their last capture event of a previous year (2008–2014). Sample sizes are shown above each bar.

**Figure 2:**
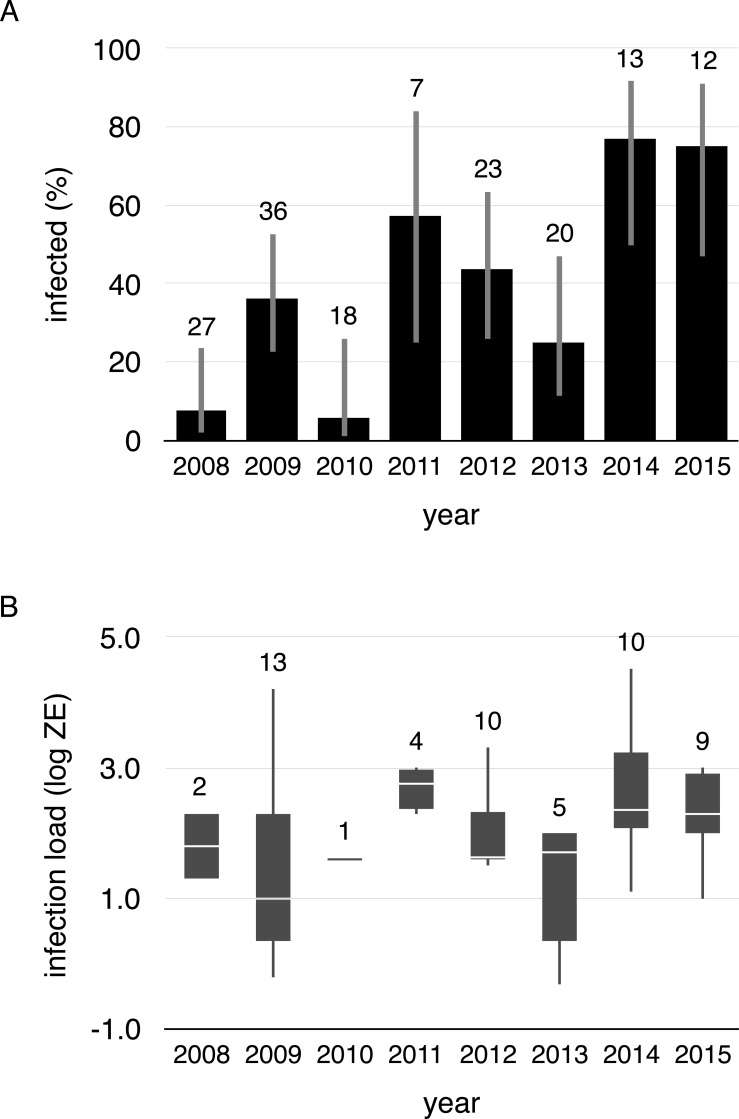
Proportion of infected toads and infection intensity during aquatic breeding seasons from 2008 to 2015. (A) Proportion of infected toads (±95% confidence intervals), and (B) Boxplot of *Bd* infection intensity (zoospore equivalents) of positive animals (boxes represent 25 and 75 percentile, the horizontal line is the median and whiskers are maximum and minimum values of infection intensity). Sample sizes are shown above each bar and boxplot.

We collected 156 swabs from the 38 toads that were captured at least twice. Fifty-four swabs tested positive for *Bd* (35%). Within-individual infections varied from year-to-year, both in terms of status and load, meaning that individuals gained and lost infections in every possible combination across years (Fig. 1, Data S1). Infection prevalence and mean intensities of breeding cohorts also differed across years (infection prevalence: *χ*^2^ = 41.19, *df* = 7, *p* < 0.001, infection intensity: *χ*^2^ = 5.4275, *df* = 1, *p* = 0.020). Infection prevalence ranged from 5% (2010) to 77% (2014) from year-to-year (mean = 41%, Fig. 2A), and *Bd* loads of infected toads averaged 1187.13 ZE (±618.08, Fig. 2B).

### Infection and activity during migration and terrestrial non-breeding seasons

Three toads shed transmitters (one male, two females) and transmitters failed to function on three toads (one male, two females). One male toad was found desiccated near LCH after the pond had completely dried, leaving 13 toads (10 male, three female) that were considered in analysis. We located those toads from 10 to 23 times after attaching transmitters (mean ± SE = 16.70 ± 1.23) and made 217 successful captures. A detailed account of the movement patterns and habitat use of these toads is provided by Daversa, Muths & Bosch (2012). Briefly, toads migrated in various directions away from ponds to inhabit rock piles and leaf litter. Individual radio-tracked toads were never found in association with other spiny common toads, though at two locations a toad was co-occupying burrow with another amphibian species (once with *Triturus marmoratus* and once with *Salamandra salamandra*). In only one instance did we observe a toad return to ponds following post-breeding migrations. Four of the 13 toads were infected when initially captured in ponds (mean ZE ± SE = 56.94 ± 31.27), all of which were males. All of the 144 swabs that we collected after toads migrated tested negative for *Bd*.

### Body temperature

Five toads out of 10 with implanted iButtons were recovered during the breeding of 2015 but iButtons failed to function on two toads. The three toads for which we recovered data came from different lakes (LP, LCH, CHM), each located in distinct basins. Components of variance in body temperature of toads, derived from a general linear model (with cubic polynomial terms for time of day and julian date; whole model *R*^2^ = 80.6%), were as follows: inter-individual = 0.3%; circadian = 1.0%; year-round = 79.3% (Table 1). Moreover, the average Pearson correlation between body temperatures of the three toads (three pairwise correlations throughout 334 common study days) were very high: *r* = 0.941 for average daily body temperature; *r* = 0.837 for minimum daily body temperature; *r* = 0.917 for maximum daily body temperature (these correlations are presented for the sake of showing the consistency-similarity in body temperature variation in the three studied toads). Thus, body temperatures of the three study toads were very similar and followed a very similar pattern of having a very low inter-individual variation with respect to time within day or year-round daily variation.

**Table 1:** General lineal mixed model analyzing the inter-individual (three different toads), circadian (time of day; 12 measurements per day every 2 h) and year-round (Julian date; 334 common study days) variation in toad body temperature. Total sample size of body temperatures is 12,264 measurements. The mixed model refers to a random intercept fixed slope model, considering the very similar pattern of body temperature variation shown by the three studied toads. SS, sums of squares; beta, standardized regression coefficients; se beta, standard error of the beta coefficients; df, degrees of freedom.

	*df*	SS	Beta	se beta
Toad	2	1,565		
Time of day				
Linear term	1	3,352	0.198	0.010
Cuadratic term	1	1,723	−0.055	0.004
Cubic term	1	2,045	−0.155	0.010
Julian date				
Linear term	1	249,862	−1.669	0.010
Cuadratic term	1	68,921	0.351	0.004
Cubic term	1	99,945	1.055	0.010
Error term	12,255	108,851		
TOTAL		560,689		

**Figure 3:**
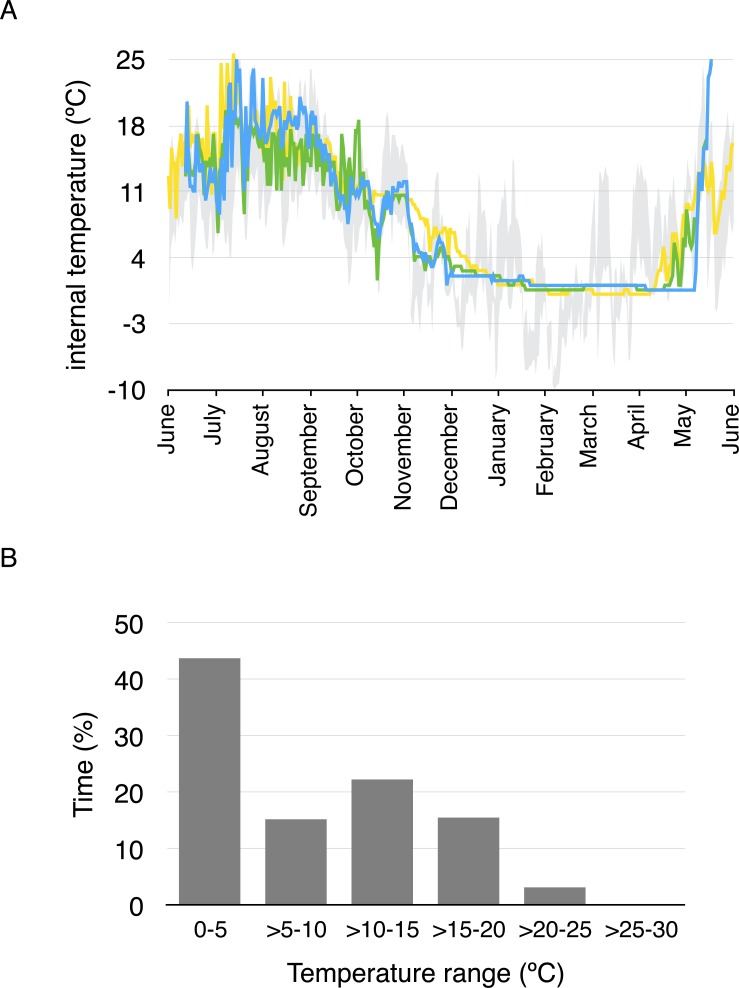
Daily average body temperature and proportion of time in a year spent within different temperature ranges for the three studied toads from whom implanted iButtons were recovered. (A) The daily average body temperature of the three toads originally captured in three different breeding ponds in Guadarrama: Laguna de Pájaros (blue line), Charca de la Mariposa (green line) and Laguna Chica (yellow line). Maximum and minimum air temperatures from the nearby meteorological station in Cotos mountain pass (1,857 m a.s.l) are shown in grey. (B) Proportion of time in a year spent within different temperature ranges for the three toads.

Average daily minimum body temperature ranged between 0.4 °C and 17 °C, while average daily maximum temperature ranged between 0.4 ° C and 25.8 °C. Body temperature was less than 5 °C for 43.4% of the year considering all temperature measurements every two hours, and less than 10 °C for 57.7% of the year (Fig. 3). Daily average body temperature of the three studied toads was below 5 °C from November 27th to April 29th, or 43.7% of the year (Fig. 3).

## Discussion

*Bd* infections in our focal host were variable across years. Infection prevalence and mean infection intensities in breeding cohorts differed across annual breeding seasons. Within-individual infections were also inconsistent across years: toads often gained and lost infections from one breeding season to the next, reflecting patterns observed in other pond-breeding amphibians, such as *Rana sierra,* that have not suffered disease-induced mass mortalities (Briggs, Knapp & Vredenburg, 2010). Annual differences in biotic (Muths, Scherer & Pilliod, 2011) and abiotic conditions (Bosch et al., in press) at breeding sites may play a role in driving this inter-annual variation in infections. At our sites, abundance of toads and sympatric hosts in breeding sites vary annually (Bosch et al., in press), as does temperature (Bosch et al., in press), and these annual differences in breeding site conditions likely affect vital epidemiological rates like host contact and zoospore accumulation in the sites.

Our radio-tracking of toads after the aquatic breeding season revealed seasonal changes in *Bd* infections that may contribute to inter-annual differences in *Bd* infections in toads. While four toads tracked in this study were infected with *Bd* at the time of capture during the aquatic breeding season, all samples collected after toads migrated away from ponds tested negative for *Bd*, and at no point during the tracking period did any detectable infections emerge in previously uninfected individuals. These findings indicate that reductions in infection prevalence in these migratory hosts are attributable to recovery and escape from infection rather than mortality of infected hosts (migratory culling), and to the best of our knowledge are one of the first direct observations of these processes. Determining the broader effects of migratory recovery and escape on seasonal *Bd* dynamics in toads will require tracking of infection and movement over a more comprehensive coverage of population, and the *Bd* detection from swabbing should be validated with other procedures (Clare et al., 2016). In addition, studies have also suggested that infections during host terrestrial phases may remain cryptic and re-emerge when animals return to water (Minting, 2012). Nevertheless, our radio-tracking indicates that post-breeding migrations pose a time constraint for infections to proliferate in individuals. If migratory recovery and escape occurs in a large proportion of the population, *Bd* dynamics across aquatic breeding seasons may be decoupled, which could also explain why infections in toads are not consistently exhibited across years. Given the typically load-dependent nature of chytridiomycosis (Vredenburg et al., 2010; Wilber et al., 2017), post-breeding migration may also modulate disease risk.

Multiple factors may result in migratory recovery. Changes in body temperature as a result of migration could play a role in migratory recovery, though at least in the three toads tracked for this study, body temperatures of toads rarely exceeded the upper thermal limits of *Bd* (26–30 °C) (Piotrowski, Annis & Longcore, 2004; Voyles et al., 2017). The largely isolated distributions of toads during and following post-breeding migrations may have been involved in the losses in infection observed, but since *Bd* infects toads via free living infective stages and is capable of re-infecting hosts via zoospore production, host-to-host transmission may not be critical for infection persistence at the individual level. We propose rather that the change from aquatic to terrestrial habitats is a key factor involved in the migratory recovery that we observed in toads. Evidence is accumulating that terrestrial habitats provide potential refuges from *Bd* (Puschendorf et al., 2011; Daversa et al., in press). For example, in another adult host species at our study sites, periodic switching from aquatic to terrestrial results in reduced proliferation and persistence of *Bd* infections (Daversa et al., in press). *Bd* can proliferate in certain terrestrial hosts (Raffel et al., 2015) and outside of hosts in certain substrates (Johnson & Speare, 2005; Kirshtein et al., 2007) however, and so the efficacy of terrestrial habitats to enable recovery from infection may depend on micro-habitat characteristics like temperature (Puschendorf et al., 2011; Raffel et al., 2015), salinity (Stockwell, Clulow & Mahony, 2015), sunlight (Puschendorf et al., 2011), and moisture retention (Johnson & Speare, 2005; Raffel et al., 2015). Although toads in our study were occasionally found in saturated terrain and damp burrows under rock piles that may contain adequate moisture levels for *Bd* (Garner et al., 2009; Raffel et al., 2015)*,* individuals were predominantly located in burrows, rock fissures, juniper bushes (*Juniperus communis nana*) and rock piles with dry sandy substrates where moisture levels were low (Daversa, Muths & Bosch, 2012), conditions which appear particularly inhibitory to *Bd* infections (Johnson & Speare, 2005; Puschendorf et al., 2011; Raffel et al., 2015).

Despite certain losses of infection during post-breeding migrations, *Bd* persists and continues to annually infect toads. Given the incomplete population coverage inherent in CMR surveys and radiotracking, toads not accounted for by surveys may sustain infections across the annual cycle. Fully aquatic larval stages of spiny toads and other sympatric species (e.g., fire salamanders, Medina et al., 2015) that overwinter in the same aquatic breeding habitats could also play a role in *Bd* maintenance across the annual cycle. With reservoir hosts to sustain a consistent pool of infective zoospores, the return of adult toads to aquatic habitats may drive forcing of infection during the breeding season. While birth pulses have explained seasonal forcing of infection in other systems (Hosseini, Dhondt & Dobson, 2004), clutches produced by toads do not hatch until later in the season after infections have accumulated in adults.

The remarkably low body temperatures of toads during the hibernation periods following post-breeding migrations may make toads more susceptible to infection when returning to breeding sites because immune function in amphibians is suppressed at low temperatures (Raffel et al., 2006). While previous work has examined effects of body temperature on *Bd* infection (Woodhams, Alford & Marantelli, 2003; Rowley & Alford, 2013; Catenazzi et al., 2017), the focus has been on effects of elevated body temperatures of hosts. Not much is known about *Bd* dynamics in hosts with body temperatures near the thermal minimum for *Bd* growth. Although the isolated and predominantly terrestrial nature of non-breeding toads makes acquisition of new infections from *Bd* exposure unlikely during hibernation, the reduced body temperatures that increase susceptibility may exacerbate seasonal forcing of infection when aquatic breeding commence. In addition, if as hypothesized a proportion of infections do remain cryptic during terrestrial phases (Minting, 2012), immunosuppression could potentially allow for infections to re-emerge before returning to breeding sites, similar to the re-activation of *Borrelia* infections in migratory birds (Glyfe et al., 2000). Owing to the limited number of the toads for which body temperature data was collected, any inferences on its broader significance for *Bd* dynamics should be made with caution. Nevertheless, the extremely low body temperatures of toads that we observed during winter months warrant further investigation of within-host dynamics of *Bd* during periods of hibernation to better understand its role in host susceptibility and seasonal *Bd* dynamics.

## Conclusions

Disentangling specific biological processes driving seasonal patterns of infection in wildlife remains a major challenge in disease ecology, in part because most studies of this topic are based on cross-sectional data and modeling. By obtaining detailed longitudinal data on host traits and infection across different seasons in the annual cycle, this study makes a step toward understanding the implications of seasonal life histories of migratory hosts for long patterns of infection in host populations. Our individual-based tracking provides direct documentation of migratory recovery. Additionally, this work empirically shows that host body temperature, a trait that affects host immunological defense, can significantly decrease during seasons of hibernation. Together, this study emphasizes that migration can alter within-host infection dynamics and also suggests a role of hibernation in seasonal infection dynamics owing to body temperature changes that decrease immune ability and thus increase susceptibility to infection.

##  Supplemental Information

10.7717/peerj.4698/supp-1 Data S1 Radiotracking, infection and internal temperature data of the studied toads For radiotracking each data point indicates a capture event. For infection data each data point indicates Bd load (zoospore equivalents) recorded for a specific year. For internal temperature data each data point indicates daily averaged internal temperature of every of the three studied toad.

## Additional Information and Declarations

### Field Study Permissions

The following information was supplied relating to field study approvals (i.e., approving body and any reference numbers): Field work was approved by the Consejería de Medio Ambiente of Madrid.

### Data Availability

The following information was supplied regarding data availability: The raw data are provided in Data S1.
